# Supplementary material for: Polyketide synthase gene clusters in the ericoid mycorrhizal fungus Oidiodendron maius: genome-wide prediction, expression profiling, and functional characterization of knockout mutants
Source: Front Plant Sci. 2026 Sep 16;17:1924430. doi: 10.3389/fpls.2026.1924430 (PMC13623903; doi:10.3389/fpls.2026.1924430)
Supplement: Supplementary file 1 [file SupplementaryFile1.zip › Supplementary Materials/Supplementary Folder 1.zip!/Oidma1_AssemblyScaffolds_Repeatmasked/knownclusterblast/cluster14/jgi.p_Oidma1_102291_mibig_hits.html]

| MIBiG Protein | Description | MIBiG Cluster | MiBiG Product | % ID | % Coverage | BLAST Score | E-value |
| --- | --- | --- | --- | --- | --- | --- | --- |
| EHA28233.1 | hypothetical\_protein | BGC0001143 | Azanigerone\_biosynthetic\_gene\_cluster | 34 | 98.191681736 | 276 | 5.8e-74 |
| BAH83502.1 | acyl-CoA\_synthetase | BGC0001253 | ACT-Toxin\_I\_biosynthetic\_gene\_cluster | 34 | 95.47920434 | 246 | 6.5e-65 |
| ADC79613.1 | BafX | BGC0000028 | Bafilomycin\_biosynthetic\_gene\_cluster | 32 | 90.7775768535 | 215 | 2.1e-55 |
| ABJ90146.1 | MoeA4 | BGC0000805 | Moenomycin\_biosynthetic\_gene\_cluster | 32 | 86.9801084991 | 208 | 1.5e-53 |
| CAE14427.1 | long-chain-fatty-acid--CoA\_ligase\_(long-chain\_acyl-CoA\_synthetase) | BGC0001180\_c2 | Isopropylstilbene\_biosynthetic\_gene\_cluster | 28 | 96.5641952984 | 203 | 8.2e-52 |
| OAQ83766.1 | phenylacetyl-\_ligase\_protein | BGC0001358 | Leucinostatins\_biosynthetic\_gene\_cluster | 36 | 64.7377938517 | 198 | 2.6e-50 |
| ADI58647.1 | AsuD3 | BGC0000187 | Asukamycin\_biosynthetic\_gene\_cluster | 31 | 85.5334538879 | 194 | 3.8e-49 |
| CAH10130.1 | feruloyl-CoA\_synthetase | BGC0000268 | Sch47554\_/\_Sch47555\_biosynthetic\_gene\_cluster | 30 | 88.4267631103 | 191 | 3.2e-48 |
| AAF08801.1 | YngI | BGC0001103 | Mycosubtilin\_biosynthetic\_gene\_cluster | 28 | 98.5533453888 | 190 | 5.5e-48 |
| AIL50189.1 | putative\_5-aminolevulinate\_CoA-ligase | BGC0000213 | Colabomycin\_biosynthetic\_gene\_cluster | 31 | 90.2350813743 | 189 | 9.4e-48 |
| ABC34346.1 | long-chain-fatty-acid--CoA\_ligase,\_putative | BGC0001102 | Malleilactone\_biosynthetic\_gene\_cluster | 29 | 98.7341772152 | 183 | 6.7e-46 |
| AGY30675.1 | Ann3 | BGC0001298 | 4-Z-annimycin\_biosynthetic\_gene\_cluster | 29 | 93.3092224231 | 182 | 1.5e-45 |
| ABS74201.1 | YngI | BGC0001095 | Fengycin\_biosynthetic\_gene\_cluster | 28 | 99.8191681736 | 181 | 2.6e-45 |
| AAX98210.1 | acyl\_CoA\_ligase | BGC0000052 | ECO-02301\_biosynthetic\_gene\_cluster | 29 | 86.4376130199 | 179 | 1.3e-44 |
| WP\_010639243.1 | 2-succinylbenzoate--CoA\_ligase | BGC0000958 | Antimycin\_biosynthetic\_gene\_cluster | 26 | 95.6600361664 | 177 | 3.7e-44 |
| AAC01710.1 | RifA | BGC0000136 | Rifamycin\_biosynthetic\_gene\_cluster | 29 | 93.8517179024 | 172 | 1.6e-42 |
| CQR60497.1 | Polyketide\_synthase,\_type\_I,\_modules:\_loading,\_1,\_2\_and\_3 | BGC0001287 | Chaxamycin\_A\_/\_chaxamycin\_B\_/\_chaxamycin\_C\_/\_chaxamycin\_D | 28 | 91.1392405063 | 165 | 1.9e-40 |
| AKA54630.1 | AMP\_dependent\_CoA-ligase | BGC0001216 | Splenocin\_biosynthetic\_gene\_cluster | 30 | 66.0036166365 | 161 | 3.6e-39 |
| AEW31026.1 | short-chain\_acyl-CoA\_synthetase | BGC0000407 | Plipastatin\_biosynthetic\_gene\_cluster | 30 | 65.6419529837 | 157 | 4e-38 |
| ABP55493.1 | thioester\_reductase\_domain | BGC0001006 | Lymphostin\_biosynthetic\_gene\_cluster | 30 | 82.4593128391 | 153 | 7.5e-37 |
| CAI94682.1 | putative\_polyketide\_synthase | BGC0000141 | Rubradirin\_biosynthetic\_gene\_cluster | 29 | 92.9475587703 | 152 | 1.3e-36 |
| ABV99085.1 | thioester\_reductase\_domain | BGC0001007 | Lymphostin\_biosynthetic\_gene\_cluster | 29 | 93.1283905967 | 151 | 2.8e-36 |
| ADM46356.1 | polyketide\_synthase | BGC0000106 | Naphthomycin\_biosynthetic\_gene\_cluster | 28 | 91.5009041591 | 150 | 6.3e-36 |
| AFU65902.1 | DacH | BGC0000216 | Dactylocycline\_biosynthetic\_gene\_cluster | 29 | 66.3652802893 | 148 | 1.8e-35 |
| AHH25585.1 | AMP-dependent\_synthetase\_and\_ligase | BGC0000957 | Ansatrienin\_(mycotrienin)\_biosynthetic\_gene\_cluster | 26 | 98.3725135624 | 148 | 1.8e-35 |
| EAL89229.1 | adenylate-forming\_enzyme,\_putative | BGC0000129 | Pyripyropene\_biosynthetic\_gene\_cluster | 29 | 71.609403255 | 145 | 2e-34 |
| ADI58632.1 | AsuB1 | BGC0000187 | Asukamycin\_biosynthetic\_gene\_cluster | 25 | 89.8734177215 | 139 | 1.1e-32 |
| AAO62588.1 | peptide\_sythetase | BGC0001016 | Microcystin\_biosynthetic\_gene\_cluster | 26 | 97.287522604 | 131 | 2.3e-30 |
| CCA65703.1 | anthranilate-CoA\_ligase | BGC0001343 | Aurachin\_biosynthetic\_gene\_cluster | 26 | 90.9584086799 | 128 | 2.6e-29 |
| AAQ84158.1 | PlmJK | BGC0000123 | Phoslactomycin\_B\_biosynthetic\_gene\_cluster | 24 | 90.2350813743 | 126 | 1.3e-28 |
| AAN32979.1 | BarE | BGC0000962 | Barbamide\_biosynthetic\_gene\_cluster | 26 | 99.095840868 | 123 | 6.3e-28 |
| CCA89328.1 | mixed\_trans-AT\_type\_I\_polyketide\_synthase/nonribosomal\_peptide\_synthetase | BGC0001111 | Rhizopodins\_biosynthetic\_gene\_cluster | 25 | 92.5858951175 | 122 | 1.8e-27 |
| BAH43766.1 | tyrocidine\_synthetase\_III | BGC0000452 | Tyrocidine\_biosynthetic\_gene\_cluster | 24 | 97.4683544304 | 121 | 3.1e-27 |
| AEU11006.1 | NpnB | BGC0001029 | Nostophycin\_biosynthetic\_gene\_cluster | 23 | 97.8300180832 | 120 | 5.4e-27 |
| AAZ03550.1 | McnA | BGC0000332 | Cyanopeptolin\_biosynthetic\_gene\_cluster | 25 | 84.2676311031 | 120 | 5.4e-27 |
| CBJ79916.1 | putative\_Ornithine\_racemase | BGC0001133 | Taxlllaid\_biosynthetic\_gene\_cluster | 23 | 94.3942133816 | 117 | 4.5e-26 |
| CBJ79915.1 | putative\_Phenylalanine\_racemase\_(ATP-hydrolyzing) | BGC0001133 | Taxlllaid\_biosynthetic\_gene\_cluster | 23 | 94.3942133816 | 117 | 4.5e-26 |
| AAO62586.1 | peptide\_sythetase | BGC0001016 | Microcystin\_biosynthetic\_gene\_cluster | 26 | 72.8752260398 | 117 | 5.9e-26 |
| ADQ55476.1 | non-ribosomal\_peptide\_synthase\_dimodule | BGC0000350 | ET-743\_biosynthetic\_gene\_cluster | 24 | 105.786618445 | 115 | 2.3e-25 |
| AFV27434.1 | indigoidine\_synthase | BGC0000375 | Indigoidine\_biosynthetic\_gene\_cluster | 24 | 81.0126582278 | 114 | 2.9e-25 |
| ABA23460.1 | Amino\_acid\_adenylation | BGC0000427 | Shinorine\_biosynthetic\_gene\_cluster | 23 | 91.3200723327 | 113 | 6.6e-25 |
| AKA54626.1 | NRPS | BGC0001216 | Splenocin\_biosynthetic\_gene\_cluster | 24 | 87.7034358047 | 112 | 1.9e-24 |
| CDG12864.1 | non-ribosomal\_peptide\_synthetase | BGC0001415 | Althiomycin\_biosynthetic\_gene\_cluster | 24 | 94.7558770344 | 111 | 2.5e-24 |
| ALK27915.1 | non-ribosomal\_peptide\_synthase | BGC0001233 | Feglymycin\_biosynthetic\_gene\_cluster | 25 | 87.5226039783 | 111 | 3.3e-24 |
| CAG23957.2 | NRPS/PKS\_protein | BGC0001089 | Bacillaene\_biosynthetic\_gene\_cluster | 25 | 98.3725135624 | 110 | 5.5e-24 |
| ALK27914.1 | non-ribosomal\_peptide\_synthase | BGC0001233 | Feglymycin\_biosynthetic\_gene\_cluster | 24 | 87.7034358047 | 109 | 1.6e-23 |
| CCJ67640.1 | TaaE | BGC0000447 | Tolaasin\_biosynthetic\_gene\_cluster | 27 | 83.9059674503 | 109 | 1.6e-23 |
| AHJ31215.1 | Long-chain-fatty-acid--CoA\_ligase | BGC0000430 | Spumigin\_biosynthetic\_gene\_cluster | 24 | 99.095840868 | 108 | 2.1e-23 |
| WP\_010639240.1 | gramicidin\_synthetase | BGC0000958 | Antimycin\_biosynthetic\_gene\_cluster | 25 | 83.7251356239 | 108 | 3.6e-23 |
| EFE73313.1 | nonribosomal\_peptide\_synthetase | BGC0000431 | Stenothricin\_biosynthetic\_gene\_cluster | 24 | 84.6292947559 | 106 | 1e-22 |
| AHI59108.1 | locillomycin\_synthase\_A | BGC0001005 | Locillomycin\_biosynthetic\_gene\_cluster | 23 | 94.2133815552 | 106 | 1.4e-22 |
| EFE73312.1 | nonribosomal\_peptide\_synthetase | BGC0000431 | Stenothricin\_biosynthetic\_gene\_cluster | 25 | 84.8101265823 | 106 | 1.4e-22 |
| AEH41793.1 | HrmO | BGC0000374 | Hormaomycin\_biosynthetic\_gene\_cluster | 26 | 88.0650994575 | 105 | 2.3e-22 |
| ABV56585.1 | KtzE | BGC0000378 | Kutznerides\_biosynthetic\_gene\_cluster | 24 | 95.8408679928 | 104 | 4e-22 |
| AAY89051.1 | hybrid\_nonribosomal\_peptide\_synthetase/polyketide\_synthase | BGC0001069 | Chivosazole\_biosynthetic\_gene\_cluster | 25 | 82.6401446655 | 104 | 5.2e-22 |
| ABD65956.1 | nonribosomal\_peptide\_synthetase | BGC0000341 | Enduracidin\_biosynthetic\_gene\_cluster | 23 | 92.5858951175 | 103 | 6.8e-22 |
| AID65222.1 | putative\_aspartate\_racemase | BGC0000335 | Cystomanamides\_biosynthetic\_gene\_cluster | 26 | 80.650994575 | 103 | 6.8e-22 |
| KPN90369.1 | NunE | BGC0001416 | Nunapeptin\_/\_nunamycin\_biosynthetic\_gene\_cluster | 22 | 84.4484629295 | 102 | 1.5e-21 |
| AAU39359.1 | lichenysin\_synthase\_LchAA | BGC0000381 | Lichenysin\_biosynthetic\_gene\_cluster | 25 | 94.3942133816 | 102 | 2e-21 |
| ADN26251.1 | ATP-dependent\_adenylase | BGC0000951 | Pacidamycin\_biosynthetic\_gene\_cluster | 24 | 88.7884267631 | 101 | 3.4e-21 |
| BAG16281.1 | putative\_2,3-dihydroxybenzoate-AMP\_ligase | BGC0000632 | Brasilicardin\_A\_biosynthetic\_gene\_cluster | 27 | 67.0886075949 | 101 | 4.4e-21 |
| CBG75492.1 | putative\_NRPS/siderophore\_biosynthesis\_protein | BGC0000423 | Scabichelin\_biosynthetic\_gene\_cluster | 24 | 91.5009041591 | 101 | 4.4e-21 |
| AHZ20774.1 | non-ribosomal\_peptide\_synthase | BGC0000369 | Hassallidins\_biosynthetic\_gene\_cluster | 22 | 84.2676311031 | 99 | 1.3e-20 |
| AEC14347.1 | nonribosomal\_peptide\_synthetase | BGC0000377 | Koranimine\_biosynthetic\_gene\_cluster | 27 | 67.6311030741 | 99 | 1.3e-20 |
| ADZ24995.1 | non-ribosomal\_peptide\_synthase/polyketide\_synthase | BGC0000380 | Leupyrrin\_biosynthetic\_gene\_cluster | 26 | 83.5443037975 | 99 | 1.3e-20 |
| BAI63288.1 | putative\_non-ribosomal\_peptide\_synthetase | BGC0000434 | SW-163\_biosynthetic\_gene\_cluster | 23 | 101.265822785 | 98 | 2.2e-20 |
| AAY93354.1 | non-ribosomal\_peptide\_synthetase\_PvdD | BGC0000413 | Pyoverdine\_biosynthetic\_gene\_cluster | 23 | 96.5641952984 | 98 | 2.2e-20 |
| AJM89738.1 | PmxE | BGC0001192 | Colistin\_biosynthetic\_gene\_cluster | 25 | 68.3544303797 | 98 | 2.2e-20 |
| CBW45647.1 | PI\_synthetase\_3\_and\_4 | BGC0000952 | Pristinamycin\_biosynthetic\_gene\_cluster | 24 | 86.2567811935 | 98 | 2.8e-20 |
| ACY06285.1 | non-ribosomal\_peptide\_synthetase | BGC0001042 | Sanglifehrin\_A\_biosynthetic\_gene\_cluster | 26 | 67.6311030741 | 97 | 3.7e-20 |
| ABD65957.1 | nonribosomal\_peptide\_synthetase | BGC0000341 | Enduracidin\_biosynthetic\_gene\_cluster | 26 | 67.4502712477 | 97 | 4.9e-20 |
| BAH43870.1 | putative\_linear\_pentadecapeptide\_gramicidin\_synthetase\_LgrB | BGC0000367 | Gramicidin\_biosynthetic\_gene\_cluster | 22 | 78.6618444846 | 97 | 4.9e-20 |
| AGE11898.1 | nonribosomal\_peptide\_synthetase | BGC0000366 | Gobichelin\_biosynthetic\_gene\_cluster | 24 | 92.4050632911 | 97 | 6.3e-20 |
| AAS98774.1 | JamA | BGC0001001 | Jamaicamide\_biosynthetic\_gene\_cluster | 24 | 101.808318264 | 97 | 6.3e-20 |
| ABD14712.1 | CesB | BGC0000320 | Cereulide\_biosynthetic\_gene\_cluster | 23 | 92.5858951175 | 96 | 8.3e-20 |
| CAQ71826.1 | non\_ribosomal\_peptide\_synthase,\_antibiotic\_synthesis;\_contains\_1\_condensation\_domain,\_1\_AMP-acid\_ligases\_II\_domain,\_PP-binding,\_Phosphopantetheine\_attachment\_site\_and\_one\_taurine\_catabolism\_dioxygenase | BGC0001189 | Taiwachelin\_biosynthetic\_gene\_cluster | 27 | 56.0578661844 | 96 | 1.1e-19 |
| AEZ51520.1 | PmxE | BGC0001153 | Polymyxin\_biosynthetic\_gene\_cluster | 25 | 68.7160940325 | 96 | 1.1e-19 |
| CCP45172.1 | Bifunctional\_enzyme\_MbtA:\_salicyl-AMP\_ligase\_(SAL-AMP\_ligase)\_+\_salicyl-S-ArCP\_synthetase | BGC0001021 | Mycobactin\_biosynthetic\_gene\_cluster | 25 | 74.1410488246 | 96 | 1.1e-19 |
| CAM56771.1 |  | BGC0000354 | Friulimicin\_biosynthetic\_gene\_cluster | 24 | 92.4050632911 | 96 | 1.4e-19 |
| EAW16180.1 | nonribosomal\_peptide\_synthase,\_putative | BGC0000293 | Acetylaszonalenin\_biosynthetic\_gene\_cluster | 27 | 71.4285714286 | 95 | 2.4e-19 |
| ALG65317.1 | Cal19 | BGC0001297 | WS9326\_biosynthetic\_gene\_cluster | 23 | 87.1609403255 | 95 | 2.4e-19 |
| BAB69700.1 | iturin\_A\_synthetase\_C | BGC0001098 | Iturin\_biosynthetic\_gene\_cluster | 23 | 97.6491862568 | 94 | 3.1e-19 |
| ABI26078.1 | OciB | BGC0000331 | Cyanopeptin\_biosynthetic\_gene\_cluster | 25 | 86.0759493671 | 94 | 4.1e-19 |
| EFL06865.1 | hypothetical\_protein | BGC0000300 | Amychelin\_biosynthetic\_gene\_cluster | 27 | 66.1844484629 | 93 | 7e-19 |
| CAJ18237.2 | non-ribosomal\_peptide\_synthetase\_B | BGC0000354 | Friulimicin\_biosynthetic\_gene\_cluster | 27 | 70.1627486438 | 93 | 9.2e-19 |
| AAY93356.2 | non-ribosomal\_peptide\_synthetase\_PvdI | BGC0000413 | Pyoverdine\_biosynthetic\_gene\_cluster | 23 | 96.2025316456 | 93 | 9.2e-19 |
| ABL74940.1 | TlmVI | BGC0001048 | Tallysomycin\_biosynthetic\_gene\_cluster | 24 | 85.1717902351 | 93 | 9.2e-19 |
| AAN74819.2 | Fum16p | BGC0000062 | Fumonisin\_biosynthetic\_gene\_cluster | 27 | 56.2386980108 | 93 | 9.2e-19 |
| AAX31555.1 | acyl-CoA\_ligase | BGC0000336 | Daptomycin\_biosynthetic\_gene\_cluster | 25 | 100.904159132 | 93 | 9.2e-19 |
| AAZ03552.1 | McnC | BGC0000332 | Cyanopeptolin\_biosynthetic\_gene\_cluster | 27 | 87.1609403255 | 92 | 1.6e-18 |
| BAH22764.1 | nonribosomal\_peptide\_synthetase | BGC0001018 | Micropeptin\_biosynthetic\_gene\_cluster | 26 | 87.7034358047 | 92 | 2e-18 |
| AAF08795.1 | MycA | BGC0001103 | Mycosubtilin\_biosynthetic\_gene\_cluster | 26 | 75.5877034358 | 92 | 2e-18 |
| CAD70195.1 | non-ribosomal\_peptide\_synthetase | BGC0001047 | Syringolin\_A\_biosynthetic\_gene\_cluster | 24 | 92.7667269439 | 92 | 2e-18 |
| CBF87069.1 | nonribosomal\_peptide\_synthase,\_putative\_(Eurofung) | BGC0001290 | Emericellamide\_A\_/\_emericellamide\_B\_biosynthetic\_gene\_cluster | 25 | 67.8119349005 | 91 | 2.7e-18 |
| ACK77757.1 | putative\_indigoidine\_synthase\_IndC | BGC0000727 | Auricin\_deoxysugar\_moieties\_biosynthetic\_gene\_cluster | 26 | 75.5877034358 | 91 | 2.7e-18 |
| ERG36782.1 | Ornithine\_racemase | BGC0000299 | Alterochromides\_biosynthetic\_gene\_cluster | 21 | 86.2567811935 | 91 | 3.5e-18 |
| ACZ65474.1 | palmitoyl-CoA\_synthetase | BGC0000140 | RK-682\_biosynthetic\_gene\_cluster | 25 | 95.2983725136 | 91 | 3.5e-18 |
| WP\_010369425.1 | ornithine\_racemase | BGC0000314 | Bromoalterochromides\_biosynthetic\_gene\_cluster | 21 | 86.2567811935 | 91 | 3.5e-18 |
| ADD82940.1 | Bat2 | BGC0001099 | Kalimantacin\_/\_batumin\_biosynthetic\_gene\_cluster | 23 | 86.6184448463 | 91 | 4.5e-18 |
| AAO62585.1 | peptide\_sythetase\_polyketide\_synthase\_fusion\_protein | BGC0001016 | Microcystin\_biosynthetic\_gene\_cluster | 26 | 69.801084991 | 91 | 4.5e-18 |
| ABD65958.1 | nonribosomal\_peptide\_synthetase | BGC0000341 | Enduracidin\_biosynthetic\_gene\_cluster | 26 | 67.9927667269 | 91 | 4.5e-18 |
| ABD14711.1 | CesA | BGC0000320 | Cereulide\_biosynthetic\_gene\_cluster | 24 | 73.9602169982 | 90 | 7.8e-18 |
| CAQ34918.1 | nonribosomal\_peptide\_synthetase/\_polyketide\_synthase | BGC0000986 | Dkxanthene\_biosynthetic\_gene\_cluster | 23 | 92.4050632911 | 89 | 1e-17 |
| ABW00331.1 | amino\_acid\_adenylation\_domain | BGC0000333 | Cyclomarin\_biosynthetic\_gene\_cluster | 25 | 89.8734177215 | 89 | 1.3e-17 |
| AIG79241.1 | Hypothetical\_protein | BGC0000419 | Ristomycin\_A\_biosynthetic\_gene\_cluster | 25 | 66.3652802893 | 89 | 1.7e-17 |
| CBF80711.1 | TdiA\_[Source:UniProtKB/TrEMBL;Acc:A7XRY0] | BGC0000442 | Terrequinone\_biosynthetic\_gene\_cluster | 26 | 69.9819168174 | 89 | 1.7e-17 |
| ADB02855.1 | AzicM | BGC0000202 | Azicemicin\_biosynthetic\_gene\_cluster | 22 | 85.8951175407 | 88 | 2.3e-17 |
| AEH41794.1 | HrmP | BGC0000374 | Hormaomycin\_biosynthetic\_gene\_cluster | 25 | 67.6311030741 | 87 | 6.6e-17 |
| ALV86866.1 | Tlo20 | BGC0001406 | Telomycin\_biosynthetic\_gene\_cluster | 24 | 87.8842676311 | 86 | 8.6e-17 |
| CBL93730.1 | NRPS | BGC0000360 | GE81112\_biosynthetic\_gene\_cluster | 24 | 66.1844484629 | 86 | 1.1e-16 |
| ALK27916.1 | non-ribosomal\_peptide\_synthase | BGC0001233 | Feglymycin\_biosynthetic\_gene\_cluster | 30 | 35.6238698011 | 86 | 1.5e-16 |
| ADD82966.1 | BatY | BGC0001099 | Kalimantacin\_/\_batumin\_biosynthetic\_gene\_cluster | 24 | 95.8408679928 | 86 | 1.5e-16 |
| AAT09805.1 | NocB | BGC0000395 | Nocardicin\_A\_biosynthetic\_gene\_cluster | 23 | 93.4900542495 | 85 | 1.9e-16 |
| ADZ24989.1 | proline\_adenylation\_protein | BGC0000380 | Leupyrrin\_biosynthetic\_gene\_cluster | 21 | 97.4683544304 | 84 | 3.3e-16 |
| AHD05679.1 | putative\_non-ribosomal\_peptide\_ligase/\_polyketide\_synthase\_hybrid | BGC0000402 | Paenilarvins\_biosynthetic\_gene\_cluster | 31 | 36.1663652803 | 84 | 4.3e-16 |
| WP\_020635019.1 | hypothetical\_protein | BGC0001211 | Albachelin\_biosynthetic\_gene\_cluster | 27 | 69.9819168174 | 84 | 4.3e-16 |
| ALV86867.1 | Tlo21 | BGC0001406 | Telomycin\_biosynthetic\_gene\_cluster | 23 | 88.2459312839 | 83 | 7.3e-16 |
| CAD29795.1 | peptide\_synthetase | BGC0001015 | Microcystin\_biosynthetic\_gene\_cluster | 25 | 68.3544303797 | 83 | 9.5e-16 |
| AGD80622.1 | AMP-dependent\_synthetase\_and\_ligase | BGC0000394 | Naphthyridinomycin\_biosynthetic\_gene\_cluster | 24 | 98.9150090416 | 82 | 1.2e-15 |
| ADJ63842.1 | non-ribosomal\_peptide\_synthetase\_protein | BGC0000424 | Serobactins\_biosynthetic\_gene\_cluster | 26 | 56.6003616637 | 82 | 1.2e-15 |
| AAG02359.1 | peptide\_synthetase\_NRPS5-4-3 | BGC0000963 | Bleomycin\_biosynthetic\_gene\_cluster | 23 | 84.6292947559 | 81 | 2.8e-15 |
| AHD05621.1 | non-ribosomal\_peptide\_ligase\_domain\_protein | BGC0001033 | Paenilamicin\_biosynthetic\_gene\_cluster | 23 | 69.801084991 | 81 | 2.8e-15 |
| CBZ42143.1 | putative\_non-ribosomal\_peptide\_synthetase | BGC0001117 | Himastatin\_biosynthetic\_gene\_cluster | 24 | 67.2694394213 | 81 | 3.6e-15 |
| CBW45640.1 | PII\_peptide\_synthetase | BGC0000952 | Pristinamycin\_biosynthetic\_gene\_cluster | 25 | 67.8119349005 | 81 | 3.6e-15 |
| ctg1\_orf000000 |  | BGC0000901 | Ferrichrome\_biosynthetic\_gene\_cluster | 29 | 48.2820976492 | 65 | 2.7e-10 |
| ACA99172.1 | Polyketide\_synthase | BGC0001160 | Nonadecene\_biosynthetic\_gene\_cluster | 26 | 101.627486438 | 79 | 1e-14 |
| AAC32048.1 | FxbC | BGC0000351 | Exochelin\_biosynthetic\_gene\_cluster | 24 | 64.5569620253 | 79 | 1.4e-14 |
| EGJ35087.1 | AMP-forming\_acyl-CoA\_synthetase | BGC0001163 | 1-heptadecene\_biosynthetic\_gene\_cluster | 24 | 105.967450271 | 76 | 8.9e-14 |
| CAC17500.1 | putative\_non-ribosomal\_peptide\_synthase | BGC0000324 | Coelibactin\_biosynthetic\_gene\_cluster | 23 | 79.5660036166 | 76 | 8.9e-14 |
| AEG64698.1 | LpmD | BGC0000379 | Laspartomycin\_biosynthetic\_gene\_cluster | 22 | 80.650994575 | 75 | 2e-13 |
| ADY16697.1 | TqaA | BGC0001142 | Tryptoquialanine\_biosynthetic\_gene\_cluster | 21 | 91.3200723327 | 75 | 2e-13 |
| AMM63162.1 | AniA | BGC0001371 | Echinocandin\_B\_biosynthetic\_gene\_cluster | 26 | 59.4936708861 | 75 | 2.6e-13 |
| AFT91378.1 | EcdA | BGC0000338 | Echinocandin\_B\_biosynthetic\_gene\_cluster | 24 | 69.6202531646 | 74 | 3.4e-13 |
| AEZ64574.1 | fatty-acid-CoA\_ligase | BGC0001065 | Herboxidiene\_biosynthetic\_gene\_cluster | 32 | 32.7305605787 | 74 | 3.4e-13 |
| CBD77732.1 | polyketide\_synthase | BGC0000974 | Crocacin\_biosynthetic\_gene\_cluster | 25 | 59.8553345389 | 74 | 4.4e-13 |
| CAD17792.1 | probable\_non\_ribosomal\_peptide\_synthetase\_protein | BGC0001363 | Ralsolamycin\_biosynthetic\_gene\_cluster | 28 | 56.0578661844 | 74 | 5.8e-13 |
| EFL06867.1 | predicted\_protein | BGC0000300 | Amychelin\_biosynthetic\_gene\_cluster | 25 | 67.8119349005 | 72 | 2.2e-12 |
| CAB38518.1 | CDA\_peptide\_synthetase\_I | BGC0000315 | Calcium-dependent\_antibiotic\_biosynthetic\_gene\_cluster | 26 | 41.9529837251 | 71 | 2.9e-12 |
| ABW71852.1 | nonribosomal\_peptide\_synthetase | BGC0000303 | Anthramycin\_biosynthetic\_gene\_cluster | 28 | 38.3363471971 | 71 | 4.9e-12 |
| ALV82356.1 | CDA\_peptide\_synthetase\_I | BGC0001370 | Lipopeptide\_8D1-1\_/\_lipopeptide\_8D1-2\_biosynthetic\_gene\_cluster | 27 | 36.8896925859 | 70 | 6.4e-12 |
| AAZ23077.1 | peptide\_synthetase | BGC0000291 | A54145\_biosynthetic\_gene\_cluster | 24 | 75.5877034358 | 70 | 6.4e-12 |
| AHH34188.1 | fatty\_acyl\_ACP\_ligase | BGC0001162 | 1-heptadecene\_biosynthetic\_gene\_cluster | 25 | 61.8444846293 | 70 | 6.4e-12 |
| AAC06347.1 | bacitracin\_synthetase\_2 | BGC0000310 | Bacitracin\_biosynthetic\_gene\_cluster | 28 | 36.3471971067 | 69 | 1.4e-11 |
| BAH23995.1 | nonribosomal\_peptide\_synthetase | BGC0000356 | Fumitremorgin\_biosynthetic\_gene\_cluster | 24 | 55.877034358 | 69 | 1.4e-11 |
| EPE34341.1 | Acetyl-CoA\_synthetase-like\_protein | BGC0001035 | Pneumocandin\_biosynthetic\_gene\_cluster | 24 | 56.0578661844 | 68 | 2.4e-11 |
| ABX37385.1 | amino\_acid\_adenylation\_domain\_protein | BGC0000984 | Delftibactin\_biosynthetic\_gene\_cluster | 34 | 28.5714285714 | 67 | 4.1e-11 |
| ctg1\_orf003 |  | BGC0000334 | Cyclosporin\_biosynthetic\_gene\_cluster | 23 | 58.4086799277 | 67 | 5.4e-11 |
| AEI70245.1 | nonribosomal\_peptide\_synthetase\_NRPS | BGC0000401 | Paenibactin\_biosynthetic\_gene\_cluster | 26 | 38.3363471971 | 66 | 9.2e-11 |
| XP\_001227984.1 | hypothetical\_protein | BGC0001240 | Serinocyclin\_A\_/\_serinocyclin\_B\_biosynthetic\_gene\_cluster | 26 | 55.1537070524 | 66 | 9.2e-11 |
| OAQ83772.1 | nonribosomal\_peptide\_synthase | BGC0001358 | Leucinostatins\_biosynthetic\_gene\_cluster | 22 | 95.6600361664 | 66 | 1.2e-10 |
| AHH34187.1 | fatty\_acyl\_ACP\_ligase | BGC0001161 | 1-heptadecene\_biosynthetic\_gene\_cluster | 32 | 31.4647377939 | 66 | 1.2e-10 |
| CAO98843.1 | hypothetical\_protein | BGC0000023 | Aurafuron\_biosynthetic\_gene\_cluster | 33 | 35.4430379747 | 66 | 1.6e-10 |
| AIW82280.1 | PuwC | BGC0001125 | Puwainaphycins\_biosynthetic\_gene\_cluster | 27 | 42.8571428571 | 65 | 2.7e-10 |
| ABS74179.1 | BmyC | BGC0001090 | Bacillomycin\_biosynthetic\_gene\_cluster | 23 | 35.9855334539 | 65 | 2.7e-10 |
| ABF88003.1 | putative\_long-chain-fatty-acid\_CoA\_ligase | BGC0000871 | VEPE\_/\_AEPE\_/\_TG-1\_biosynthetic\_gene\_cluster | 28 | 45.2079566004 | 64 | 3.5e-10 |
| EAL89049.1 | nonribosomal\_peptide\_synthase,\_putative | BGC0000355 | Fumiquinazolines\_biosynthetic\_gene\_cluster | 23 | 69.9819168174 | 63 | 7.8e-10 |
| CAA16183.1 | polyketide\_synthase | BGC0001063 | Undecylprodigiosin\_biosynthetic\_gene\_cluster | 30 | 44.122965642 | 63 | 7.8e-10 |
| AFV96135.1 | AMP-dependent\_synthetase/ligase | BGC0001064 | Cylindrocyclophane\_biosynthetic\_gene\_cluster | 28 | 42.4954792043 | 63 | 7.8e-10 |
| CDG76957.1 | non-ribosomal\_peptide\_synthetase | BGC0000446 | Tilivalline\_biosynthetic\_gene\_cluster | 21 | 84.0867992767 | 63 | 1e-09 |
| AHF22854.1 | MarL | BGC0000091 | Marineosin\_biosynthetic\_gene\_cluster | 30 | 35.0813743219 | 63 | 1e-09 |
| ADN13832.1 | Beta-ketoacyl\_synthase | BGC0001164 | 1-heptadecene\_biosynthetic\_gene\_cluster | 28 | 43.5804701627 | 62 | 1.7e-09 |
| AAY37650.1 | Amino\_acid\_adenylation | BGC0000437 | Syringomycin\_biosynthetic\_gene\_cluster | 23 | 33.8155515371 | 62 | 2.3e-09 |
| AEZ53969.1 | putative\_AMP-binding\_domain-containing\_protein | BGC0000144 | Salinomycin\_biosynthetic\_gene\_cluster | 32 | 25.8589511754 | 62 | 2.3e-09 |
| AGA37269.1 | NRPS | BGC0000819 | Paraherquamide\_biosynthetic\_gene\_cluster | 23 | 60.9403254973 | 61 | 3e-09 |
| ABF87031.1 | non-ribosomal\_peptide\_synthetase/polyketide\_synthase | BGC0000393 | Myxoprincomide\_biosynthetic\_gene\_cluster | 29 | 35.8047016275 | 61 | 5e-09 |
| AEP18656.1 | WAPS1 | BGC0000461 | WAP-8294A2\_(lotilibcin)\_biosynthetic\_gene\_cluster | 27 | 35.4430379747 | 60 | 6.6e-09 |
| BAO73252.1 | non-ribosomal\_peptide\_synthetase | BGC0000456 | Verlamelin\_biosynthetic\_gene\_cluster | 24 | 51.7179023508 | 60 | 8.6e-09 |
| CDG17987.1 | Putative\_Ornithine\_racemase\_(fragment) | BGC0000464 | Xenoamicins\_biosynthetic\_gene\_cluster | 25 | 33.9963833635 | 60 | 8.6e-09 |
| AAF08797.1 | MycC | BGC0001103 | Mycosubtilin\_biosynthetic\_gene\_cluster | 22 | 35.4430379747 | 59 | 1.5e-08 |
| ABV56588.1 | KtzH | BGC0000378 | Kutznerides\_biosynthetic\_gene\_cluster | 27 | 40.5063291139 | 58 | 2.5e-08 |
| AHB82069.1 | non\_ribosomal\_peptide\_synthetase | BGC0001231 | Microsclerodermins\_biosynthetic\_gene\_cluster | 27 | 33.0922242315 | 58 | 3.3e-08 |
| AMB48444.1 | long-chain-fatty-acid--CoA\_ligase | BGC0001357 | Carbamidocyclophane\_biosynthetic\_gene\_cluster | 26 | 35.9855334539 | 57 | 4.3e-08 |
| AGN74885.1 | nonribosomal\_peptide\_synthetase | BGC0000459 | Griseoviridin\_/\_viridogrisein\_biosynthetic\_gene\_cluster | 25 | 33.6347197107 | 57 | 7.3e-08 |
| AHN85651.1 | Phn2 | BGC0000122 | Phenylnannolone\_A\_biosynthetic\_gene\_cluster | 27 | 30.3797468354 | 56 | 9.5e-08 |
| BAB69698.1 | iturin\_A\_synthetase\_A | BGC0001098 | Iturin\_biosynthetic\_gene\_cluster | 29 | 37.9746835443 | 56 | 1.6e-07 |
| CBW54660.1 | Acyl-CoA\_ligase | BGC0000971 | Cinnabaramide\_biosynthetic\_gene\_cluster | 27 | 44.3037974684 | 56 | 1.6e-07 |
| CDG17985.1 | Putative\_Ornithine\_racemase\_(fragment) | BGC0000464 | Xenoamicins\_biosynthetic\_gene\_cluster | 25 | 35.8047016275 | 55 | 2.8e-07 |
| ABS74181.1 | BmyA | BGC0001090 | Bacillomycin\_biosynthetic\_gene\_cluster | 29 | 32.7305605787 | 54 | 3.6e-07 |
| AED90004.1 | non-ribosomal\_peptide\_synthetase\_ThaC1 | BGC0000443 | Thanamycin\_biosynthetic\_gene\_cluster | 22 | 34.7197106691 | 54 | 6.2e-07 |
| CAQ18831.1 | nonribosomal\_polypetide\_synthetase | BGC0000954 | Ajudazol\_biosynthetic\_gene\_cluster | 23 | 36.7088607595 | 52 | 1.4e-06 |
| AAY93355.1 | non-ribosomal\_peptide\_synthetase\_PvdJ | BGC0000413 | Pyoverdine\_biosynthetic\_gene\_cluster | 31 | 21.157323689 | 51 | 4e-06 |
